# Association of the variants in the *BUD13-ZNF259* genes and the risk of hyperlipidaemia

**DOI:** 10.1111/jcmm.12291

**Published:** 2014-04-30

**Authors:** Lynn Htet Htet Aung, Rui-Xing Yin, Dong-Feng Wu, Wei Wang, Cheng-Wu Liu, Shang-Ling Pan

**Affiliations:** a Department of Cardiology, Institute of Cardiovascular Diseases, The First Affiliated Hospital, Guangxi Medical UniversityNanning, Guangxi, China; b Department of Pathophysiology, School of Premedical Sciences, Guangxi Medical UniversityNanning, Guangxi, China

**Keywords:** hyperlipidaemia, BUD13 homolog (BUD13), ZNF259 zinc finger protein 259 (ZNF259), genetic polymorphisms

## Abstract

The single nucleotide polymorphisms (SNPs) in the BUD13 homolog (BUD13) and zinc finger protein 259 (ZNF259) genes have been associated with one or more serum lipid traits in the European populations. However, little is known about such association in the Chinese populations. Our objectives were to determine the association of the *BUD13*/*ZNF259* SNPs and their haplotypes with hypercholesterolaemia (HCH)/hypertriglyceridaemia (HTG) and to identify the possible gene–gene interactions among these SNPs. Genotyping of 6 SNPs was performed in 634 hyperlipidaemic and 547 normolipidaemic participants. The *ZNF259* rs2075290, *ZNF259* rs964184 and *BUD13* rs10790162 SNPs were significantly associated with serum lipid levels in both HCH and non-HCH populations (*P* < 0.008–0.001). On single locus analysis, only *BUD13* rs10790162 was associated with HCH (OR: 2.23, 95% CI: 1.05, 4.75, *P* = 0.015). The G-G-A-A-C-C haplotype, carrying rs964184-G-allele, was associated with increased risk of HCH (OR: 1.35, 95% CI: 1.10, 1.66, *P* = 0.005) and HTG (OR: 1.75, 95% CI: 1.39, 2.21, *P* *=* 0.000). The A-C-G-G-C-C and A-C-A-G-T-C haplotypes, carrying rs964184-C-allele, were associated with reduced risk of HCH (OR: 0.77, 95% CI: 0.61, 0.99, *P* = 0.039 and OR: 0.66, 95% CI: 0.47, 0.94, *P* *=* 0.021 respectively). On multifactor dimensionality reduction analyses, the two- to three-locus models showed a significant association with HCH and HTG (*P* < 0.01–0.001). The *BUD13/ZNF259* SNPs, which were significant in the European populations, are also replicable in the Southern Chinese population. Moreover, inter-locus interactions may exist among these SNPs. However, further functional studies are required to clarify how these SNPs and genes actually affect the serum lipid levels.

## Introduction

Derangement in lipid metabolism can have many unfavourable consequences, of which, coronary artery disease (CAD) is one of the most disastrous ones and contributes to the major disease burden worldwide [1–3]. It is well accepted that the serum lipid level is modulated by genetic and environmental factors [4]. Several studies have proved that the heritability of lipid trait is ∼40–70% and genetic factors are the major determinant of the familial resemblance in plasma lipids and lipoproteins [4–6]. Therefore, identification of the genes involved in lipid metabolism could provide a clue to search for novel pathway in lipid regulation and thereby new therapeutic or preventive methods for CAD. With the rapid progress in genome-wide association studies (GWAS), a great number of lipid-related loci have been discovered [7–9]. As the frequency of the susceptible alleles and their effect size on serum lipid levels may vary across world populations or between hyperlipidaemic and normolipidaemic populations, replication of GWAS signals in different ethnic groups and assessment of their association with the risk of hyperlipidaemia have become fundamental in validation of these signals [10,11].

Recently, several single nucleotide polymorphisms (SNPs) in the BUD13 homolog (BUD13; Gene ID: 84811; HGNC: 28199) and zinc finger protein 259 (ZNF259; Gene ID: 8882; OMIM: 603901) genes nearby *APOA5* have been associated with one or more serum lipid traits [12–15]. The BUD13/ZNF259 genes are located on chromosome 11q23.3 and encode for BUD13 homolog protein and zinc finger protein (ZPR1) respectively. BUD13 is one of the subunits of the RES complex, which was previously identified in yeast as a splicing factor that affects nuclear pre-mRNA retention [16]. ZNF259, also known as ZPR1, is an essential regulatory protein for normal nuclear function in cell proliferation and signal transduction [17,18]. The promoter site of *ZNF259* was bounded by the proxisome proliferator-activated receptor gamma (PPARG) 1 and 2, which play an important role in insulin sensitivity and obesity [19,20], and also bounded by the hepatocyte nuclear factor 4 alpha (HNF4α, nuclear receptor 2A1), which is known to activate a variety of genes involved in glucose, fatty acid and cholesterol metabolism in the liver, kidney, intestine and pancreas [21]. Although the association of the *BUD13/ZNF259* SNPs and serum lipid levels has been reported in the European and Asian Indian populations, little is known about such association in the Chinese populations. In addition, the association of their haplotypes and possible gene–gene interaction among these SNPs with the risk of hyperlipidaemia has never been detected before. Therefore, this study was performed (*i*) to assess the association of the *BUD13* 237 + 1741T>C (rs10790162), 323-575A>G (rs17119975), *147C>T (rs11556024), 64G>T (rs35585096) and *ZNF259* 1093-336G>A (rs2075290) and *365 + 359C>G (rs964184) SNPs and serum lipid levels in individuals with hypercholesterolaemia (HCH)/hypertriglyceridaemia (HTG); (*ii*) to evaluate the association of these SNPs and their haplotypes with the risk of HCH/HTG; and (*iii*) to identify the possible gene–gene interactions among these SNPs.

## Materials and methods

### Study populations

The participants were recruited from Luocheng Mulao Autonomous County, Guangxi Zhuang Autonomous Region, People's Republic of China in 2010. A total of 1181 participants were randomly selected from our stratified, randomized cluster samples [22]. There were 634 hyperlipidaemic (TC >5.17 mmol/l and/or TG >1.70 mmol/l) and 547 normolipidaemic (TC ≤5.17 mmol/l and TG ≤1.70) individuals, aged 18–80 years. The age and gender distribution were matched between the two populations. The participants with a history of cardiovascular disease including CAD and stroke, diabetes, chronic illness including cardiac, renal, thyroid problems and/or a history of taking lipid-modulating medications such as statins or fibrates were excluded. Within the hyperlipidaemic population to assess the association of SNPs with risk of HCH and HTG separately, the hyperlipidaemic populations were subdivided into hypercholesterolaemic (TC >5.17 mmol/l) and hypertriglyceridaemic (TG >1.70 mmol/l) groups. Informed consents were obtained from all participants after they have received a full explanation of the study. The study was reviewed and approved by the Ethics Committee of the First Affiliated Hospital, Guangxi Medical University.

### Epidemiological survey and biochemical measurements

The epidemiological survey was carried out by using internationally standardized methods and following a common protocol [23]. Information on demographics, socioeconomic status, lifestyle, past medical history and family disease history was collected by using standardized questionnaires [22]. The intake of alcohol was quantified as the number of liangs (about 50 g) of rice wine, corn wine, rum, beer or liquor consumed during the preceding 12 months. Alcohol consumption was categorized into groups of grams of alcohol per day: 0 (non-drinkers), ≤25 and >25. Smoking status was categorized into the groups of cigarettes per day: 0 (non-smokers), ≤20 and >20. The methods of blood pressure, height, weight and waist circumference measurements have been described in the previous studies [24,25]. Fasting venous blood samples were taken and the levels of serum total cholesterol (TC), TG, high-density lipoprotein cholesterol (HDL-C), and low-density lipoprotein cholesterol (LDL-C) in the samples were directly determined by enzymatic methods with commercially available kits, Tcho-1, TG-LH (RANDOX Laboratories Ltd., Ardmore, Diamond Road, Crumlin Co. Antrim, UK), Cholestest N HDL, and Cholestest LDL (Daiichi Pure Chemicals Co. Ltd., Tokyo, Japan) respectively. Serum apolipoprotein (Apo) A1 and ApoB levels were assessed by the immunoturbidimetric assay by using a commercial kit (RANDOX Laboratories Ltd.). All determinations were performed with an autoanalyzer (Hitachi Ltd., Tokyo, Japan). The normal values of serum TC, TG, HDL-C, LDL-C, ApoA1 and ApoB levels and the ratio of ApoA1 to ApoB in our Clinical Science Experiment Centre were 3.10–5.17, 0.56–1.70, 1.16–1.42, 2.70–3.10 mmol/l, 1.20–1.60, 0.80–1.05 g/l and 1.00–2.50 respectively [24,25].

### SNP selection and genotyping

We selected six SNPs in the *BUD13/ZNF259* with the following assumptions: (*i*) Tag SNPs, which were established by Haploview (Broad Institute of MIT and Harvard, USA, version 4.2) or functional SNPs in functional areas of the gene fragments (http://www.ncbi.nlm.nih.gov/SNP/snp); (*ii*) a known minor allele frequency higher than 1% in European ancestry from the Human Genome Project Database; and (*iii*) the target SNP region should be adequately replicated by PCR, and the polymorphic site should have a commercially available restriction endonuclease enzyme cleavage site to be genotyped with the restriction fragment length polymorphism (RFLP).

Genomic DNA was isolated from peripheral blood leucocytes using the phenol-chloroform method [24,25]. Genotyping of six SNPs was performed by PCR and RFLP. The characteristics of each SNP and the details of each primer pair, annealing temperature, length of the PCR products and corresponding restriction enzyme used for genotyping are summarized in Tables S1 and S2. The PCR products of the samples (two samples of each genotype) were sequenced with an ABI Prism 3100 (Applied Biosystems, International Equipment Trading Ltd., Vernon Hills, IL, USA) in Shanghai Sangon Biological Engineering Technology & Services Co. Ltd., China. The sequencing results of each genotype sequence were directly submitted to GenBank's Gene Expression Omnibus database. The GenBank accession numbers for the DNA sequences of each genotype are described in Table S3.

### Statistical analysis

The statistical analyses were performed with the statistical software package SPSS 17.0 (SPSS Inc., Chicago, IL, USA). The quantitative variables were presented as the mean ± SD for those, that are normally distributed, and the medians and interquartile ranges for TG, which is not normally distributed. General characteristics between the two groups were compared by the Student's unpaired *t*-test. The allele frequency and genotype distribution, as well as haplotype frequency between the groups was analysed by the chi-squared test; and the Hardy–Weinberg equilibrium was verified with the standard goodness-of-fit test. Pair-wise linkage disequilibria and haplotype frequencies among the SNPs were analysed using Haploview (Broad Institute of MIT and Harvard, USA, version 4.2). The association between genotypes and serum lipid parameters was tested by ancova. Any variants associated with the serum lipid parameter at a value of *P* < 0.008 (corresponding to *P* < 0.05 after adjusting for six independent tests by the Bonferroni correction) were considered statistically significant. Unconditional logistic regression was used to assess the correlation between the risk of hyperlipidaemia and genotypes (*ZNF259* rs2075290: GG = 1, AG/AA = 2; *ZNF259* rs964184: CC = 1, CG/GG = 2; *BUD13* rs10790162: GG = 1, GA/AA = 2; *BUD13* rs17119975: AA = 1, AG/GG = 2; *BUD13* rs11556024: CC = 1, CT/TT = 2; *BUD13* rs35585096: GG = 1, GT =2). Age, sex, body mass index (BMI), smoking and alcohol consumption were adjusted for the statistical analysis. Two-sided *P* < 0.05 was considered statistically significant.

The inter-locus interaction was analysed by generalized multifactor dimensionality reduction (GMDR) method, using GMDR software. The cross-validation consistency score provides the degree of consistency when the selected interaction is identified as the best model among all possibilities considered. The testing balanced accuracy provides the degree of interaction, which accurately predicts the case–control status with scores between 0.50 (indicating that the model predicts no better than the chance) and 1.00 (indicating perfect prediction). A sign test or a permutation test provides *P*-value for predicting accuracy to measure the significance of an identified model. The best model is selected as the combination of marker with maximum cross-validation consistency and minimum prediction error.

## Results

### Characteristics of the studied populations

Tables 1 and 2 compare the general characteristics and serum lipid levels between the HCH and non-HCH populations and between the HTG and non-HTG populations respectively. Both HCH and HTG individuals had significantly higher anthropometric parameters than their control individuals (*P* < 0.05–0.001). The age and gender distribution, and the% of participants who smoked and consumed alcohol were significantly different between the HTG and non-HTG populations (*P* < 0.05–0.001); however, no such difference was noted between the HCH and non-HCH populations (*P* > 0.05).

**Table 1:** Anthropometric and metabolic characteristics between the hypercholestrolaemic and non-hypercholestrolaemic individuals

Characteristics	Cases (*n* = 557)	Controls (*n* = 547)	*P*-value
Age (years)	51.41 ± 14.38	50.80 ± 15.67	0.499*
Male, *n* (%)	227 (40.8)	220 (40.2)	0.856†
Height (cm)	154.44 ± 8.00	154.28 ± 7.50	0.354*
Weight (kg)	54.25 ± 9.10	52.32 ± 9.20	<0.001*
Body mass index (kg/m^2^)	22.76 ± 3.61	21.74 ± 3.04	<0.001*
Waist circumference (cm)	76.30 ± 8.20	74.40 ± 8.29	<0.001*
Systolic blood pressure (mmHg)	131.10 ± 20.38	127.77 ± 20.48	0.007*
Diastolic blood pressure (mmHg)	82.99 ± 11.72	80.40 ± 11.21	<0.001*
Pulse pressure (mmHg)	48.11 ± 15.30	47.37 ± 15.40	0.424*
Cigarette smoking, *n* (%)			
Non-smoker	421 (75.6)	415 (75.9)	0.537†
≤20 Cigarette smoking/day	103 (18.5)	92 (16.8)	
>20 Cigarette smoking/day	33 (5.9)	40 (7.3)	
Alcohol consumption, *n* (%)			
Non-drinker	425 (76.3)	440 (80.4)	0.245†
≤25 g/day	52 (9.3)	41 (7.5)	
>25 g/day	80 (14.4)	66 (12.1)	
Blood glucose level (mmol/l)	6.10 ± 1.83	5.90 ± 1.41	0.040*
Total cholesterol (mmol/l)	5.99 ± 0.81	4.31 ± 0.78	<0.001*
Triglyceride (mmol/l)	1.32 (0.99)	0.94 (0.64)	<0.001*
HDL-C (mmol/l)	1.66 ± 0.53	1.86 ± 0.41	<0.001*
LDL-C (mmol/l)	3.62 ± 0.77	2.42 ± 0.53	<0.001*
Apolipoprotein (Apo) A1 (g/l)	1.24 ± 0.34	1.44 ± 0.34	<0.001*
ApoB (g/l)	1.09 ± 0.43	0.82 ± 0.46	<0.001*
ApoA1/Apo B	1.45 ± 0.53	1.73 ± 0.78	<0.001*

* Comparison between cases and controls by *t*-test.

† Comparison between cases and controls by chi-squared test.

^‡^Comparison between cases and controls by non-parametric test. The values of triglyceride were presented as median (interquartile range).

**Table 2:** Anthropometric and metabolic characteristics between the hypertriglyceridaemic and non- hypertriglyceridaemic individuals

Characteristics	Cases (*n* = 254)	Controls (*n* = 850)	*P*-value
Age (years)	48.34 ± 13.92	51.5 ± 15.41	0.003*
Male, *n* (%)	124 (48.8)	350 (41.2)	0.031†
Height (cm)	157.42 ± 7.68	154.28 ± 8.97	<0.001*
Weight (kg)	59.08 ± 9.88	51.54 ± 8.40	<0.001*
Body mass index (kg/m^2^)	23.81 ± 3.41	21.62 ± 2.97	<0.001*
Waist circumference (cm)	80.63 ± 8.63	73.72 ± 7.61	<0.001*
Systolic blood pressure (mmHg)	132.31 ± 19.71	127.71 ± 20.71	0.003*
Diastolic blood pressure (mmHg)	84.25 ± 11.67	80.52 ± 11.35	<0.001*
Pulse pressure (mmHg)	48.06 ± 14.35	47.19 ± 15.63	0.459*
Cigarette smoking, *n* (%)			
Non-smoker	155 (61.0)	664 (78.1)	<0.001†
≤20 Cigarette smoking/day	81 (31.9)	160 (18.8)	
>20 Cigarette smoking/day	18 (7.1)	26 (3.1)	
Alcohol consumption, *n* (%)			
Non-drinker	148 (58.3)	643 (75.6)	<0.001†
≤25 g/day	28 (11.0)	61 (7.2)	
>25 g/day	78 (30.7)	146 (17.2)	
Blood glucose level (mmol/l)	6.15 ± 2.11	5.93 ± 1.43	0.040*
Total cholesterol (mmol/l)	5.58 ± 1.29	4.83 ± 1.06	<0.001*
Triglyceride (mmol/l)	2.41 (1.60)	0.91 (0.51)	<0.001*
HDL-C (mmol/l)	1.49 ± 0.38	1.80 ± 0.50	<0.001*
LDL-C (mmol/l)	3.07 ± 1.02	2.86 ± 0.82	0.006*
Apolipoprotein (Apo) A1 (g/l)	1.30 ± 0.37	1.32 ± 0.35	0.488*
ApoB (g/l)	1.08 ± 0.49	0.88 ± 0.44	<0.001*
ApoA1/Apo B	1.36 ± 0.67	1.68 ± 0.69	<0.001*

* Comparison between cases and controls by *t*-test.

† Comparison between cases and controls by chi-squared test.

^‡^Comparison between cases and controls by non-parametric test. The values of triglyceride were presented as median (interquartile range).

### Genotype and allele frequencies

Tables 3 and 4 describe the genotype and allele frequencies of the *BUD13/ZNF259* SNPs. The genotype distribution of all six SNPs agreed with Hardy–Weinberg equilibrium (*P* > 0.05 for all). The genotype frequency of the *BUD13* rs10790162 SNP and the allele frequencies of the *ZNF259* rs964184, *BUD13* rs10790162 and *BUD13* rs17119975 SNPs were significantly different between the HCH and non-HCH populations (*P* < 0.05–0.01). On the other hand, the genotype and allele frequencies of the *ZNF259* rs2075290, *ZNF259* rs964184 and *BUD13* rs10790162 SNPs and the allele frequency of the *BUD13* rs11556024 SNP were significantly different between the HTG and non-HTG groups (*P* < 0.001 for each). A significant linkage disequilibrium (LD) was noted among the *ZNF259* rs2075290, *ZNF259* rs964184 and *BUD13* rs10790162 SNPs (*r*^2^ > 0.5, *P* < 0.001).

**Table 3:** The association between the *BUD13/ZNF259* polymorphisms with hypercholesterolaemia

		Hypercholesterolaemia				
				
SNP	Genotype	Genotype distribution, *n* (%)			OR (95%CI)	*P*-value
				
		Cases (*n* = 557)	Controls (*n* = 547)	*P*-value		
*ZNF259* 1093-336G>A rs2075290	AA	268 (48.1)	296 (54.1)		1.22 (0.74, 2.02)	0.437
	AG/GG	289 (59.8)	251 (45.9)	0.135		
	MAF	333 (29.9)	288 (26.3)	0.062		
*ZNF259* *365 + 359C>G rs964184	CC	309 (55.5)	335 (61.2)		0.69 (0.33, 1.47)	0.340
	CG/GG	248 (34.5)	212 (38.8)	0.097		
	MAF	286 (25.7)	238 (21.8)	0.030		
*BUD13* 237 + 1741T>C rs10790162	GG	340 (62.2)	307 (56.1)		2.23 (1.05, 4.75)	0.015
	GA/AA	217 (37.8)	240 (44.0)	0.025		
	MAF	234 (21.4)	294 (26.4)	0.006		
*BUD13* 323-575A>G rs17119975	AA	381 (68.4)	339 (62.0)		0.95 (0.69, 1.31)	0.743
	AG/GG	176 (28.4)	208(38.0)	0.078		
	MAF	194 (17.4)	231 (21.1)	0.027		
*BUD13* *147C>T rs11556024	CC	480 (86.2)	458 (83.7)		0.92 (0.61, 1.39)	0.683
	CT/TT	77 (13.8)	89 (16.3)	0.472		
	MAF	78 (7.0)	91 (8.0)	0.245		
*BUD13* 64G>T rs35585096	CC	548 (98.4)	541 (98.9)		0.76 (0.21, 2.75)	0.676
	CA	9 (1.6)	6 (1.1)	0.457		
	MAF	9 (0.8)	6 (0.6)	0.451		

MAF, minor allele frequency; ZNF259, zinc finger protein 259; BUD, BUD13 homolog.

**Table 4:** The association between the *BUD13/ZNF259* polymorphisms with hypertriglyceridaemia

		Hypertriglyceridaemia				
				
SNP	Genotype	Genotype distribution, *n* (%)			OR (95% CI)	*P*-value
				
		Cases (*n* = 254)	Controls (*n* = 850)	*P*-value		
*ZNF259* 1093-336G>A rs2075290	AA	103 (40.6)	461 (54.2)		1.17 (0.77, 1.76)	0.466
	AG/GG	151 (59.4)	389 (45.8)	0.000		
	MAF	181 (35.2)	440 (25.9)	0.000		
*ZNF259* *365 + 359C>G rs964184	CC	122 (48.0)	522 (61.4)		0.78 (0.42, 1.46)	0.437
	CG/GG	132 (52.0)	328 (38.6)	0.000		
	MAF	156 (30.8)	368 (21.7)	0.000		
*BUD13* 237 + 1741T>C rs10790162	GG	118 (46.5)	529 (62.2)		1.38 (0.73, 2.6)	0.322
	GA/AA	136 (53.5)	321 (37.8)	0.000		
	MAF	165 (32.5)	363 (21.4))	0.000		
*BUD13* 323-575A>G rs17119975	AA	174 (68.5)	546 (64.2)		0.78 (0.60, 1.02)	0.064
	AG/GG	80 (31.5)	304 (35.8)	0.210		
	MAF	85 (16.7)	340 (20.0)	0.101		
*BUD13* *147C>T rs11556024	CC	220 (86.6)	718 (84.5)		0.85 (0.61, 1.19)	0.339
	CT/TT	34 (13.4)	132 (15.5)	0.402		
	MAF	68 (13.4)	135 (7.9)	0.000		
*BUD13* 64G>T rs35585096	CC	251 (98.8)	838 (98.6)		1.37 (0.48, 3.91)	0.551
	CA	3 (1.2)	12 (1.4)	0.781		
	MAF	6 (1.2)	24 (1.4)	0.861		

### Genotypes and serum lipid levels

**Fig. 1:**
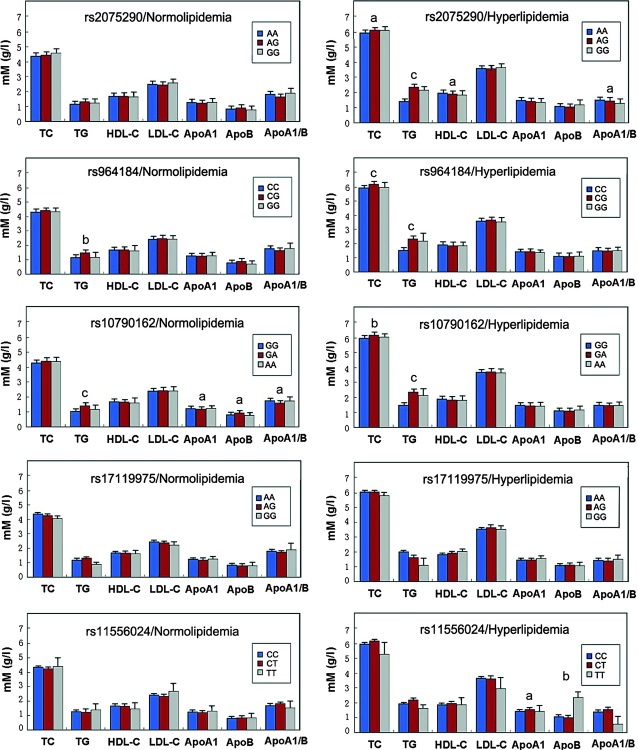
The genotypes of 5 *BUD13/ZNF259* SNPs and serum lipid levels in the hyperlipidaemic and normolipidaemic populations. TC: total cholesterol; TG: triglyceride; HDL-C: high-density lipoprotein cholesterol; LDL-C: low-density lipoprotein cholesterol; ApoA1: apolipoprotein A1; ApoB: apolipoprotein B; ApoA1/B: the ratio of apolipoprotein A1 to apolipoprotein B. ^a^*P* < 0.05, ^b^*P* < 0.01 and ^c^*P* < 0.001.

**Fig. 2:**
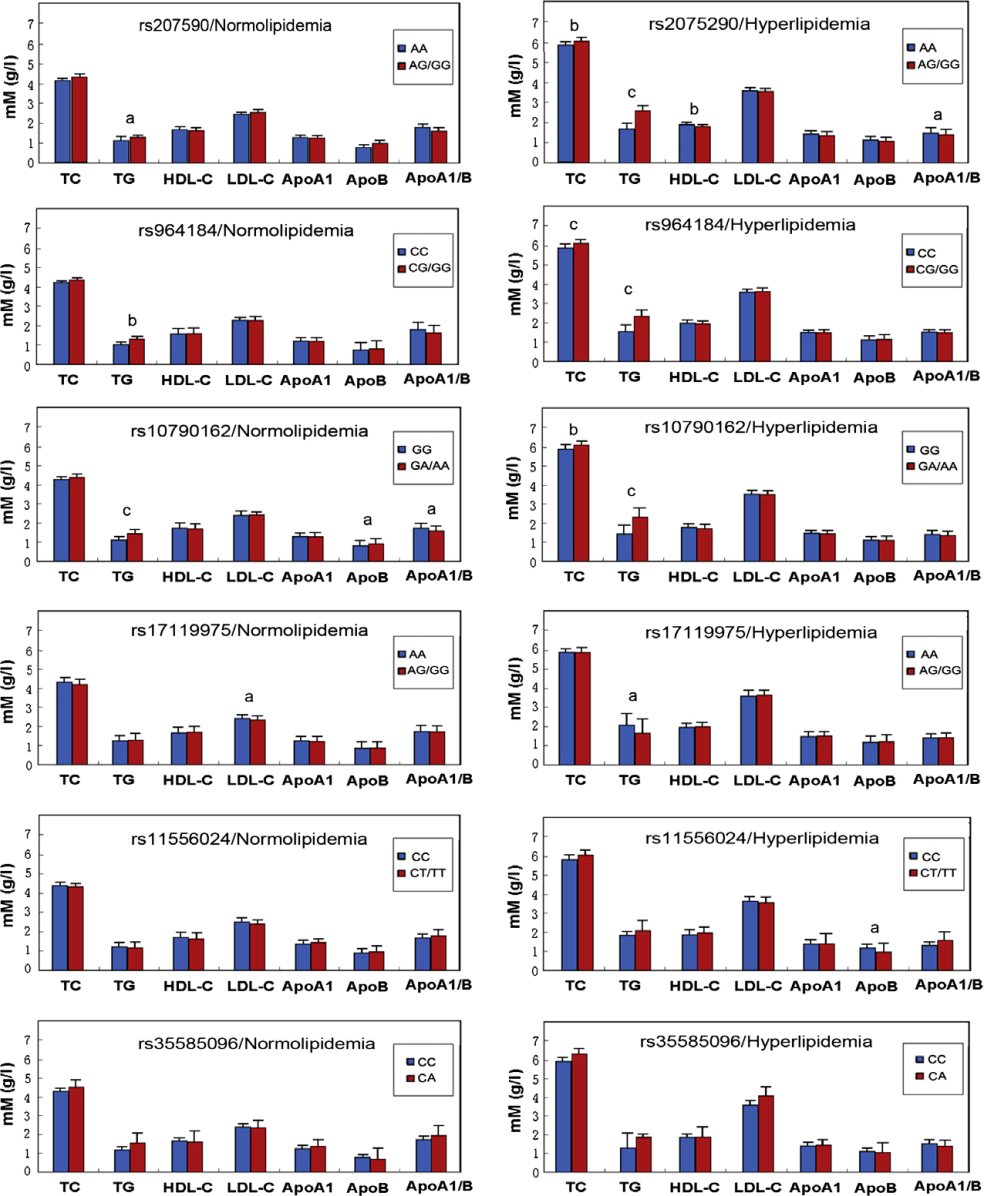
The genotypes of 6 *BUD13/ZNF259* SNPs (in additive model) and serum lipid levels in the hyperlipidaemic and normolipidaemic populations. TC: total cholesterol; TG: triglyceride; HDL-C: high-density lipoprotein cholesterol; LDL-C: low-density lipoprotein cholesterol; ApoA1: apolipoprotein A1; ApoB: apolipoprotein B; ApoA1/B: the ratio of apolipoprotein A1 to apolipoprotein B. ^a^*P* < 0.05, ^b^*P* < 0.01 and ^c^*P* < 0.001.

Figure 1 depicts the association between the genotypes and serum lipid levels in the hyperlipidaemic and normolipidaemic populations. After the Bonferroni correction of *P*-values, the levels of TG (*ZNF259* rs964184 and *BUD13* rs10790162) in the normolipidaemic individuals were different between the three genotypes (*P* < 0.008–0.001); whereas the levels of TC (*ZNF259* rs964184 and *BUD13* rs10790162), TG (*ZNF259* rs2075290, *ZNF259* rs964184 and *BUD13* rs10790162) and ApoB (*BUD13* rs11556024) in the hyperlipidaemic population were different between the three genotypes (*P* < 0.008–0.001). When the minor homozygous genotype was combined with the heterozygous genotype to enhance power (Fig. 2), the levels of TG (*ZNF259* rs964184 and *BUD13* rs10790162) in the hyperlipidaemic population were different between the genotypes (*P* < 0.008–0.001); whereas, the levels of TC (*ZNF259* rs2075290, *ZNF259* rs964184 and *BUD13* rs10790162), TG (*ZNF259* rs2075290, *ZNF259* rs964184 and *BUD13* rs10790162) and HDL-C (*ZNF259* rs2075290) in the hyperlipidaemic population were different between the genotypes (*P* < 0.008–0.001).

After adjusting age, gender, BMI, smoking and alcohol consumption, logistic regression analysis showed that the *BUD13* rs10790162 SNP was associated with HCH with an odd ratio (OR) of 2.23 (95% CI: 1.05, 4.75, *P* = 0.015). The remaining five SNPs did not exhibit any significant association with either HCH or HTG (Tables 3 and 4).

### Haplotypes and the risk of hyperlipidaemia

As shown in Table 5, the haplotype of A-C-A-G-C-C (in the order of the *ZNF259* rs2075290, *ZNF259* rs964184, *BUD13* rs10790162, *BUD13* rs17119975, *BUD13* rs11556024 and *BUD13* rs35585096 SNPs) was the commonest haplotype and represented ∼50% of the sample. The haplotype of G-G-A-A-C-C, carrying rs964184-G-allele, was associated with increased risk of HCH (OR: 1.35, 95% CI: 1.10, 1.66, *P* = 0.005) and HTG (OR: 1.75, 95% CI: 1.39, 2.21, *P* = 0.000). The haplotypes of A-C-G-G-C-C and A-C-A-G-T-C, carrying rs964184-C-allele, were associated with reduced risk of HCH (OR: 0.77, 95% CI: 0.61, 0.99, *P* = 0.039 and OR: 0.66, 95% CI: 0.47, 0.94, *P* = 0.021 respectively).

**Table 5:** The association between the *BUD13/ZNF259* haplotypes and hypercholesterolaemia/hypertriglyceridaemia

Haplotypes	Hypercholesterolaemia				Hypertriglyceridaemia			
		
	Cases, *n* (%)	Control, *n* (%)	OR (95% CI)	*P*-value	Cases, *n* (%)	Control, *n* (%)	OR (95% CI)	*P*-value
A-C-A-G-C-C	548.03 (0.49)	525.94 (0.48)	1.09 (0.92, 1.30)	0.328	220.88 (0.44)	850.41 (0.50)	0.81 (0.66, 1.00)	0.050
G-G-A-A-C-C	262.83 (0.24)	209.02 (0.19)	1.35 (1.10, 1.66)	0.005	143.23 (0.28)	327.17 (0.19)	1.75 (1.39, 2.21)	<0.001
A-C-G-G-C-C	136.38 (0.12)	169.78 (0.16)	0.77 (0.61, 0.99)	0.039	57.56 (0.11)	249.67 (0.15)	0.77 (0.57, 1.05)	0.099
A-C-A-G-T-C	55.42 (0.05)	81.50 (0.07)	0.66 (0.47, 0.94)	0.021	25.41 (0.05)	116.09 (0.07)	0.75 (0.48, 1.16)	0.193
G-C-G-G-C-C	30.71 (0.03)	47.43 (0.04)	0.64 (0.40, 1.01)	0.054	16.08 (0.03)	65.81 (0.04)	0.84 (0.45, 1.47)	0.548
Rare Hap (<0.03)	80.61 (0.07)	60.34 (0.06)	–	–	44.84 (0.09)	90.15 (0.05)	–	–

### Gene–gene interactions for hyperlipidaemia

Table 6 shows the impacts of combination among the *BUD13*/*ZNF259* SNPs, which were analysed by GMDR. The two- and three-locus models showed a significant association with the risk of HCH and HTG (*P* < 0.01–0.001). The three-locus model was chosen as the best one, owing to the fact of having the highest level of testing accuracy (58% and 59% respectively for HCH and HTG) and good cross-validation consistency (8/10).

**Table 6:** Best inter-locus interaction models identified by the generalized multifactor dimensionality reduction method

Locus no	Best combination for HTC	Cross-validation consistency	Testing accuracy	*P*-value
2	rs17119975 rs11556024	6/10	0.5631	0.0107
3	rs2075290 rs17119975 rs11556024	8/10	0.5764	0.0010

Locus no	Best combination for HTG	Cross-validation consistency	Testing accuracy	*P*-value
2	rs17119975 rs10790162	7/10	0.5810	0.0010
3	rs2075290 rs17119975 rs10790162	8/10	0.5870	0.0010

HTC: hypercholesterolaemia; HTG: hypertriglyceridaemia.

## Discussion

The main findings of the present study encompass (*i*) the associations of the *BUD13/ZNF259* SNPs with serum lipid levels in individuals with HCH/HTG; (*ii*) the correlation of these SNPs and their haplotypes with HCH and HTG; and (*iii*) possible gene–gene interaction among these SNPs to influence HCH/HTG. To the best of our knowledge, this is the first report on the inter-locus interaction among *BUD13/ZNF259* SNPs in the Chinese population. Of six SNPs examined, the *BUD13* rs35585096 SNP, which has not been genotyped by the International HapMap Consortium, was found to have lesser than 1% in the Chinese population. The observed allele frequencies of remaining five SNPs in the non-HCH/non-HTG populations were consistent with those of the International HapMap Chinese Han Beijing samples (http://hapmap.ncbi.nlm.nih.gov/cgi-perl/gbrowse/hapmap27_B36/).

A couple of years ago, Johansen *et al*. found that the individuals with *ZNF259* rs964184-G-allele were associated with 3.28-folds increased in the risk of HTG in the European population and 1.8-folds increased in the risk of HTG in the Mexican population [26]. This minor G-allele was also associated with CAD risk in the American, European and Han Chinese populations [27,28]. Likewise, in a European population's large-scale association study, Schunkert *et al*. found that the G-allele carriers had 1.13 times increased in the risk of CAD than the G-allele non-carriers [29]. In addition, Kathiresan *et al*. estimated that the individuals who carried the risk G-allele could increase ∼0.3 SD in serum TG and decrease 0.17 SD in serum HDL-C [15]. Moreover, in a large-scale GWAS, this variant was associated with quantitative change in serum lipid levels; in particular, the G-allele carriers were associated with increased serum TC for 4.68 (4.11–5.25) mg/dl, TG for 16.95 (16.01–17.89) mg/dl and LDL-C for 2.85 (2.32–3.38) mg/dl and decreased HDL-C for 1.5 (1.28–1.72) mg/dl as compared with the G-allele non-carriers. The same direction of association was seen in the subsequent replication study comprising three non-European (East Asians, South Asians and African-Americans) populations [12]. In another replication study of GWAS-derived lipid genes in Asian Indians, Braun *et al*. concluded that among six previously genome-wide significant SNPs on 11q23.3 region, rs964184 SNP was the strongest signals for TG [30]. However, a GWAS of combined white European and Asian Indian populations showed that the G allele was associated with decreased HDL-C only but not with TG [13]. In the present study, we found that the *ZNF259* rs964184-G-allele was more frequent in HCH/HTG than non-HCH/non-HTG populations. The rs964184 minor-G-allele carriers tend to have higher TC (in the both hyperlipidaemic and normolipidaemic populations) and higher TG levels (in the hyperlipidaemic population) than the minor allele non-careers. However, no association with serum HDL-C levels was noted in our study populations. The reason for this discrepancy among these studies is not fully understood. It might be because of the difference in the genetic background, ethnic difference in LD pattern and/or difference in the environmental factors [31,32].

In a recent meta-analysis, comprising 13 independent European ancestry studies, the SNPs of *ZNF259* rs2075290 and *BUD13* rs10790162 were identified as the top association SNPs for metabolic syndrome [33]. Notably, the minor allele of each SNP was associated with 0.39 (0.29, 0.49) and 0.38 (0.28, 0.48) unit increased in pleiotropic effects (associated simultaneously with more than two individual traits) of HDL-C and TG, 0.41 (0.31, 0.51) and 0.39 (0.29, 0.49) unit increased in pleiotropic effects of waist circumference and TG respectively [33]. Partially consistent to previous study, our findings showed that the rs2075290 minor-A-allele carriers had higher TG levels (in the both hyperlipidaemic and normolipidaemic populations) and higher TC, TG and lower HDL-C levels (in the hyperlipidaemic population) than the minor-A-allele non-careers. Similarly, the individuals with rs10790162-A allele were found to have higher TC (in the hyperlipidaemic population) and TG levels (in the both hyperlipidaemic and normolipidaemic populations) than the minor A-allele non-careers. However, since we did not analyse the pleiotropic effect in our study, we were unable to verify such pleiotropic effects here.

When assessing the association of *BUD13/ZNF259* SNPs and the risk of hyperlipidaemia, this study showed that although the *ZNF259* rs964184 and *ZNF259* rs2075290 SNPs were strongly associated with serum lipid levels in the both hyperlipidaemic and normolipidaemic populations, these variants did not reach statistically significant association with HCH/HTG risk. Only the *BUD13* rs10790162 (which is in moderate LD with *ZNF259* rs964184 and *ZNF259* rs2075290) SNP achieved significant association with the risk of HCH/HTG. In addition, we noticed that the haplotype of G-G-A-A-C-C, carrying *ZNF259* rs964184-G-allele, was associated with increased risk of HCH and HTG. The haplotypes of A-C-G-G-C-C and A-C-A-G-T-C, carrying *ZNF259* rs964184-C-allele, were associated with reduced risk of HCH. These findings were supported by the Timothy's study, which assessed the haplotypes among rs735041, rs180326, rs964184, rs618923, rs110047459 and rs533556 SNPs in Asian Indians. Timothy *et al*. found that two haplotypes carrying risk G-allele were associated with raised TG and haplotype carrying protective C-allele was associated with reduced TG [30] and concluded that rs964184 SNP had a putative role on affecting TG concentration independently [30].

On GMDR analyses, an inter-locus interaction among the *ZNF259* rs2075290, *BUD13* rs17119975 and *BUD13* rs11556024 SNPs was found to be associated with the risk of HCH and that among the *ZNF259* rs2075290, *BUD13* rs17119975 and *BUD13* rs10790162 SNPs were associated with the risk of HTG, respectively. In single locus analysis, only one SNP was associated to the risk of HTG. However, in multi-locus (GMDR) analyses, a significant association with HCH and HTG was found in two- to three-locus models. These findings indicate that a potential gene–gene interaction might exist among the *BUD13/ZNF259* SNPs. Unfortunately, no previous study has investigated the inter-locus interaction between these SNPs, and therefore we cannot make comparisons with our results. Although, a statistically significant SNP–SNP interaction was noted in this study, the biological mechanism underlying these genes and their interactions is still yet to be defined.

The BUD13 and ZNF259 genes are located in the downstream of the APOA5 gene and therefore many studies predicted that their association with serum lipid levels might be related to the nearby APO5 gene. The *APOA5*, which encodes the liver-expressed ApoA5, is a /crucial determinant of TG rich lipoprotein metabolism in both mice and humans. Plasma ApoA5 is associated with chylomicrons, very low-density lipoprotein (VLDL), and HDL at very low concentrations; on the other hand, *APOA5* retained in an intrahepatic pool is associated with lipid droplets. Plasma ApoA5 affects the distribution of ApoC3 molecules on VLDL and thus promotes lipolysis. ApoA5 is regulated by several transcription factors that are involved in plasma lipoprotein and glucose homoeostasis. PPARα agonists, such as fibrates, induce *APOA5* expression in humans. In mice, overexpression of *APOA5* markedly decreased plasma TG concentration, whereas mice lacking the *APOA5* became severely hypertriglyceridaemic; however, the mechanism of *APOA5*'s regulation and its molecular function were incompletely understood [34]. Taken all of these facts into consideration, it is possible that the significant SNPs identified in the *BUD13/ZNF259* region might be in high LD with some of the functional SNPs in *APOA5*, which is known to affect the lipid metabolism. Thus, an in-depth study of the biological actions of these genes is crucial to clarify which SNPs are functional and how these genes actually affect the serum lipid levels. It is expected that the physiological function of *BUD13/ZNF259* will be elucidated in a not too distant future.

## Study limitations

There are several potential limitations in our study. First, the number of participants available for minor allele of some SNPs was not high enough to calculate a strong power. Hence, further studies with larger sample size are needed to confirm our results. Second, we were unable to alleviate the effect of diet during the statistical analysis. Third, although we have detected the interactions of the *BUD13-ZNF259* SNPs and hyperlipidaemia in this study, many unmeasured environmental and genetic factors still need to be considered. Besides, the interactions of gene–environment and environment–environment on serum lipid levels remain to be determined. For the clear understanding of biological mechanism underlying hyperlipidaemia, an enormous amount of common variants with small effects and rare variants with large effects still remain to be determined.

## Conclusions

Our study confirmed that the genetic variants, which were significant in the European populations, are also replicable in the Southern Chinese hyperlipidaemic and normolipidaemic populations. The haplotype of G-G-A-A-C-C, carrying rs964184-G-allele, is associated with increased risk of HCH and HTG. The haplotypes of A-C-G-G-C-C and A-C-A-G-T-C, carrying rs964184-C-allele, are associated with reduced risk of HCH and HTG. In addition, possible inter-locus interactions among the *BUD13/ZNF259* SNPs are also noted. However, further functional studies of these genes are still required to clarify which SNPs are functional and how these genes actually affect the serum lipid levels.

## Supporting information

**Table S1.** Characteristics of the BUD13/ZNF259 SNPs. **Table S2.** The sequences of forward and backward primers and restriction enzymes for genotyping of the BUD13/ZNF259 SNPs. **Table S3.** The GenBank accession numbers for the DNA sequences of each genotype.
