# Supplementary material for: Association of the variants in the BUD13-ZNF259 genes and the risk of hyperlipidaemia
Source: J Cell Mol Med. 2014 Apr 30;18(7):1417–28. doi: 10.1111/jcmm.12291 (PMC4124025; doi:10.1111/jcmm.12291)
Supplement: Supplementary file 1 [file jcmm0018-1417-SD1.doc]

**Supplemental Table 1. Characteristics of the *BUD13/ZNF259* SNPs.**

| **SNP ID (rs#)** | **HUGO name** | **Chromosome** | **Position** | **Gene region** | **Minor allele frequencies ^a^** |
| --- | --- | --- | --- | --- | --- |
| *ZNF259* |  |  |  |  |  |
| rs2075290 | NM_003904.3:c.1093-336G>A | 11 | 116653296 | Intron2 | 25.6% |
| rs964184 | NM_003904.3:c.*365+359C>G | 11 | 116648917 | nearGene- 3 | 21.9% |
| *BUD13* |  |  |  |  |  |
| rs10790162 | NM_001159736.1:c.237+1741T>C | 11 | 116639104 | Intron2 | 23.3% |
| rs17119975 | NM_001159736.1:c.323-575A>G | 11 | 116634557 | Intron3 | 25.5% |
| rs11556024 | NM_001159736.1:c.*147C>T | 11 | 116619051 | UTR-3 | 12.5% |
| rs35585096 | [NM_001159736.1:c.64G>T](http://www.ncbi.nlm.nih.gov/nuccore/NT_033899.8?report=graph&db=nucleotide&v=20205982:20206082&content=5&m=20206033!&mn=rs35585096) | 11 | 116643617 | mRNA | Not yet reported |

nearGene- 3: 0.5 kb away from gene near 3’; UTR-3: 3’ untranslated region; **^a^** Han Chinese Bejing from International HapMap Project.

**Supplemental Table 2. The sequences of forward and backward primers and restriction enzymes for genotyping of the *BUD13/ZNF259*** SNPs.

| **SNP** | **Primer sequence** | **Annealing temperature** | **PCR product** | **Restriction enzyme/site** | **Restriction fragment (bp)** | **All-ele** |
| --- | --- | --- | --- | --- | --- | --- |
| [*ZNF259*](http://www.ncbi.nlm.nih.gov/entrez/query.fcgi?db=gene&cmd=Retrieve&dopt=Graphics&list_uids=8882) |  |  |  |  |  |  |
| rs2075290 | GAGTCTATGGGATAATGTGC | 56.7 °C | 331bp | SspI* | 331 | G |
|  | AATTCCAGGCCTAACTTTGG |  |  | AAT^ATT | 151+180 | A |
| rs964184 | CCCTGCTTTACATTCCTCCA | 58 °C | 496bp | Bsp143I (Sau3AI) * | 496 | C |
|  | CCTCACCCTCCTTCCACATA |  |  | GA^TC | 56+440 | G |
| [*BUD13*](http://www.ncbi.nlm.nih.gov/entrez/query.fcgi?db=gene&cmd=Retrieve&dopt=Graphics&list_uids=84811) |  |  |  |  |  |  |
| rs10790162 | CTAAAGCGAACCATACCCTTTG | 52 °C | 530bp | TaaI(HpyCH4III)Bst4C** | 357+173 | A |
|  | AGGGAGCCAATTTAGTTTTGGT |  |  | ACN^GT | 260+173+97 | G |
| rs17119975 | AACTATTTAATCCATTTCACG(T)GTGC^x^ | 59.5 °C | 358bp | BshNI (BanI) * | 358 | A |
|  | ATTTCATTCTGTGGTGCCTTTGA |  |  | G^GYRCC | 21+ 337 | G |
| rs11556024 | CAAACCCTGGTCAATGAGAAA | 58.5 °C | 572bp | TaqI ** | 572 | T |
|  | TATTAGGCAGGCTGGTCTTGA |  |  | T^CGA | 103+469 | C |
| rs35585096 | CCAAGAGGGAAGGAACTGCC | 61.5°C | 463bp | NaeI* | 103+360 | A |
|  | AGAAGAAACCTGGTGGCGTCG |  |  | GCC^GGC | 103+294+66 | C |

*, Restriction endonuclease enzyme digestion occurred at 37°C; **, enzyme digestion occurred at 65°C. **X**, mismatching nucleotides (T was modified to G) in the forward primer, BshNI (BanI) could find the restriction site (5'... G/GYRCC...3') in the amplicon sequence.

**Supplemental Table 3. The GenBank accession numbers for the DNA sequences of each genotype.**

| **SNP** | **Genotype** | **GeneBank accession number** |
| --- | --- | --- |
| *ZNF259* rs2075290 | AA/ AG/ GG | KF306313 - 306315 |
| *ZNF259* rs964184 | CC/ CG/ GG | KF306310 - 306312 |
| *BUD13* rs10790162 | GG / GA/ AA | KF306302 - 306304 |
| *BUD13* rs17119975 | AA/ AG/ GG | KF306316 - 306318 |
| *BUD13* rs11556024 | CC/ CT/ TT | KF306305 - 306307 |
| *BUD13* rs35585096 | CC/ CA | KF306308/ KF306309 |

MLXIPL: MLX interacting protein-like, BUD13: BUD13 homolog and ZNF259: zinc finger protein 259.
